# Eco-Functional PVDF Mixed Matrix Membranes: Characterization and Regeneration in Natural Rubber Skim Latex Purification

**DOI:** 10.3390/polym18080925

**Published:** 2026-04-10

**Authors:** Rianyza Gayatri, Rendy Muhamad Iqbal, Wirach Taweepreda, Muzafar Zulkifli, Ahmad Naim Ahmad Yahaya

**Affiliations:** 1 Department of Chemical Engineering, Faculty of Engineering, Universitas Sriwijaya, Jalan Palembang-Prabumulih KM 32, Indralaya 30662, Indonesia; rianyzagayatri@ft.unsri.ac.id; 2 Department of Chemistry, Faculty of Mathematics and Natural Sciences, Universitas Palangka Raya, Palangka Raya 73111, Indonesia; iqbal.rm@mipa.upr.ac.id; 3 Polymer Science Program, Division of Physical Science, Faculty of Science, Prince of Songkla University, Hat-Yai 90110, Thailand; wirach.t@psu.ac.th; 4 Green Chemistry and Sustainability Cluster, Branch Campus Malaysian Institute of Chemical and BioEngineering Technology, Universiti Kuala Lumpur, Alor Gajah 78000, Melaka, Malaysia; muzafar@unikl.edu.my

**Keywords:** skim latex, natural rubber, composite membranes, PVDF, SDS cleaning, fouling

## Abstract

Concentrated natural rubber skim latex is a sustainable, value-added product derived from natural rubber latex processing, offering high rubber content, fine particle size, and shorter polymer chains compared to pure latex, making it suitable for diverse industrial applications. This study employed an environmentally friendly ultrafiltration method using composite membranes composed of polyvinylidene fluoride (PVDF), titanium dioxide (TiO_2_), and polyvinylpyrrolidone (PVP) to concentrate skim latex without hazardous chemicals. The process generated two fractions: concentrated skim latex and skim serum. Membrane performance and fouling behavior were evaluated using FESEM-EDX and FTIR. Post-filtration analysis revealed significant latex particle deposition on the membrane surface, with elemental mapping confirming the presence of organic and inorganic residues. FTIR spectra indicated interaction between latex components and membrane functional groups, though the membrane’s structural integrity remained intact. Sodium dodecyl sulfate (SDS) was assessed as a cleaning agent and demonstrated the effective partial restoration of membrane performance, as confirmed by flux recovery (PVDF-PVP-TiO_2_ membrane recovered to a slightly higher flux of 7.35 L/m^2^h). These results highlight the membrane’s durability, fouling characteristics, and cleaning potential, supporting its reusability in latex processing. This study contributes to the development of sustainable separation technologies in the rubber industry, promoting circular economy and zero-discharge practices.

## 1. Introduction

In the pursuit of sustainable and environmentally responsible manufacturing processes, the natural rubber industry has increasingly focused on the efficient utilization of its by-products. The dry rubber content of field latex is in the range of 30 to 45% [1]. In particular, skim latex, a residual stream generated during the production of natural rubber latex, has garnered attention due to its potential for transformation into high-value materials [2,3]. Through the application of advanced separation technologies, this by-product can be refined into concentrated skim latex, a material characterized by a high rubber content, smaller particle size, and shorter polymer chains compared to standard natural rubber latex [4]. These properties lend concentrated skim latex enhanced suitability for a variety of downstream industrial applications while simultaneously supporting zero-discharge objectives and contributing to a circular economy model.

One of the most promising approaches for processing skim latex into a concentrated form is the use of ultrafiltration membrane separation technology [5]. To fully utilize by-products from natural rubber latex processing, skim latex has to undergo a pressure-driven membrane separation process [3]. This method offers a clean and energy-efficient alternative to traditional chemical-intensive processes, enabling the selective retention of rubber fractions while allowing aqueous components (skim serum) to permeate through the membrane [6,7,8]. Notably, this approach eliminates the need for harmful chemical reagents, aligning with green processing principles. Despite the growing adoption of membrane technologies in rubber latex processing, the majority of recent studies have focused on optimizing operating parameters and latex concentration efficiencies, rather than investigating the behavior and characteristics of the membranes themselves, particularly after exposure to skim latex streams, which are known to induce fouling phenomena.

Fouling remains a significant challenge in membrane separation applications [9,10]. During filtration, the accumulation of organic and inorganic substances on the membrane surface or within its pores can drastically reduce permeability, alter membrane morphology, and degrade separation performance [9]. In the context of skim latex filtration, fouling can be particularly pronounced due to the presence of rubber particles, proteins, lipids, and other colloidal components [10]. Understanding the fouling propensity of membranes following natural rubber skim latex separation is therefore crucial for the development of long-lasting, efficient filtration systems [1].

In this study, a composite or a mixed matrix membrane (MMM) composed of polyvinylidene fluoride (PVDF), titanium dioxide (TiO_2_), and polyvinylpyrrolidone (PVP) is employed for the ultrafiltration of skim latex [11,12]. PVDF is well known for its mechanical robustness and chemical resistance [13,14], TiO_2_ contributes hydrophilicity and potential antifouling properties [14], and PVP acts as a pore-forming agent enhancing the membrane’s structural characteristics [15]. The synergistic combination of these materials is expected to improve both filtration performance and fouling resistance.

To assess the structural and chemical changes in the membrane after filtration, comprehensive membrane characterization is conducted using Scanning Electron Microscopy (SEM) to examine surface morphology, Fourier-Transform Infrared (FTIR) Spectroscopy to identify chemical alterations, and Atomic Force Microscopy (AFM) to investigate changes in surface roughness and topography. These techniques provide multi-scale insight into membrane behavior before and after latex exposure, offering valuable information on fouling mechanisms and membrane integrity.

Furthermore, a critical aspect of this work is the evaluation of membrane cleaning and reusability. Specifically, the effectiveness of sodium dodecyl sulfate (SDS), a commonly used anionic surfactant, is examined as a cleaning agent for restoring membrane performance. Sodium dodecyl sulfate (SDS), also referred to as sodium lauryl sulfate, is a widely utilized anionic surfactant comprising a 12-carbon hydrophobic alkyl chain linked to a negatively charged sulfate group [16]. Due to its strong surface-active properties, SDS serves as a primary ingredient in a broad range of household and personal care formulations, including detergents, dishwashing agents, hand cleansers, and cosmetic products [17].

Cleaning efficacy is quantified by measuring the skim serum flux before and after SDS treatment across multiple cycles. This assessment is essential to determine whether SDS can effectively remove foulants from the membrane surface, thus extending the membrane’s operational life and maintaining high filtration efficiency. In addition to analyzing membrane performance, this study also compares the physicochemical properties of raw skim latex and concentrated skim latex obtained via ultrafiltration. The comparison in this study aims to highlight the transformation and potential value-added applications of the concentrated product, supporting the broader objective of enhancing resource efficiency in natural rubber processing.

Ultimately, this research seeks to fill critical knowledge gaps in the literature by characterizing the membrane after skim latex filtration, investigating fouling behavior and recovery potential through cleaning, and contributing to the development of more sustainable, reusable membrane systems for industrial latex processing. The findings are expected to provide key insights for the scale-up and practical implementation of membrane technologies in rubber industries aiming for eco-innovation and zero-waste production models.

## 2. Materials and Methods

### 2.1. Materials

Polyvinylidene fluoride (PVDF) was procured from Arkema Inc. (Philadelphia, PA, USA). N,N-Dimethylacetamide (DMAc) was obtained from Merck (Rahway, NJ, USA), and used as the solvent. Titanium dioxide (TiO_2_) nanoparticles were supplied by Evonik GmbH (Essen, Germany). Polyvinylpyrrolidone (PVP) was procured from Sigma-Aldrich (St. Louis, MI, USA), which also provided sodium dodecyl sulfate (SDS) for membrane cleaning experiments. The PVDF concentration was 16 wt.%, PVP at 1 wt.% and TiO_2_ at 2 wt%, in accordance with previously reported procedures [11].

### 2.2. Methods

#### 2.2.1. Natural Rubber Skim Latex Ultrafiltration Process and Membrane Cleaning

A total of 1.5 L of skim latex volume was used for the filtration process, which was continuously recirculated within the ultrafiltration system [5]. The transmembrane pressure (TMP) was maintained at 1 bar, consistent with previous studies. Upon the completion of each ultrafiltration run, the concentrated retentate was returned to the feed tank to maintain a constant feed volume throughout the process [18]. After each operation, the fouled membrane was thoroughly cleaned to evaluate the distinct contributions of external and internal fouling. In the typical cleaning experiments and fouling mitigation, the fouled membrane was chemically cleaned in a two-step method. Firstly, the membrane was soaked in a 10% sodium dodecyl sulfate (SDS) solution for one hour to facilitate the chemical reaction with the layer of foulant. The membrane in the ultrafiltration system was rinsed with deionized water for a duration of 30 min. Then, skim latex filtration was conducted, and flux was remeasured for a two-hour filtration time.

#### 2.2.2. Membrane Performance Analysis

The membranes were fabricated using the phase inversion method, as described in previous research [11]. The performance of a membrane is typically evaluated based on its selectivity and flux rate. Flux is defined as the volumetric flow rate of liquid passing through a membrane per unit area over a specified period [19]. Flux rates were determined using an ultrafiltration experimental setup. Skim serum filtrate samples were measured every 15 min for one hour to determine the skim latex flux. The flux (J) was measured using Equation (1) as reported by [20]:

(1) $$J=\frac{Q}{A\;\times \;t}$$

J is the skim latex flux (L/m^2^h), Q is the permeate volume in liters, t is the time in hours needed to achieve volume Q, and A stands for the effective membrane area in square meters.

#### 2.2.3. Membrane Post-Filtration Characterization

The ultrafiltration of natural rubber skim latex using mixed matrix membranes (MMMs) yields two distinct product streams: the skim latex concentrate (retentate), enriched with rubber content, and the skim serum (permeate), primarily composed of water and low-molecular-weight solutes. To evaluate membrane behavior and structural changes induced by the filtration process, detailed membrane characterization is conducted before and after use. A Field Emission Scanning Electron Microscope and energy-dispersive X-ray (SEM-EDX) are employed to examine surface morphology, conduct elemental analysis, and visualize any fouling or pore blockage that may occur during operation, while Fourier-Transform Infrared (FTIR) Spectroscopy provides insights into potential chemical interactions and modifications in functional groups on the membrane surface. These complementary analyses are essential for understanding membrane fouling mechanisms, evaluating structural integrity, and informing strategies for membrane cleaning and reuse.

## 3. Results and Discussion

### 3.1. Membrane Characterization After Natural Rubber Skim Latex Filtration

#### 3.1.1. Fourier-Transform Infrared (FTIR) Spectroscopy of Membranes

**Figure 1:**
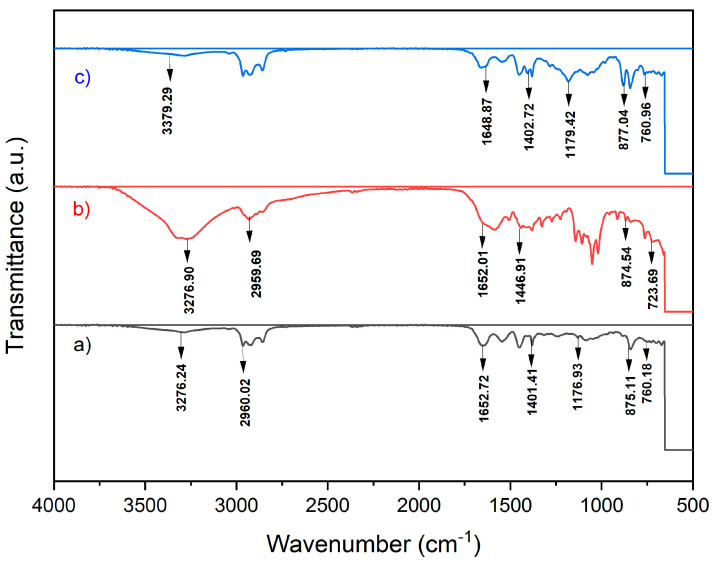
(**a**) FTIR spectroscopy of PVDF–PVP–TiO_2_ membrane after skim latex filtration, (**b**) FTIR spectroscopy of PVDF–PVP–TiO_2_ membrane after skim latex filtration and after cleaning using SDS, (**c**) FTIR spectroscopy of pristine PVDF membrane after skim latex filtration.

The FTIR spectra of PVDF pristine and composite membranes are shown in Figure 1 following the filtration of skim latex for two hours. The comparative FTIR spectra of the three membranes indicate that the PVDF–PVP–TiO_2_ membrane maintains its structural integrity throughout the skim latex filtration and subsequent SDS cleaning processes. The presence of characteristic peaks for PVDF (CF_2_ stretching and bending) and PVP (C=O and C–N stretching) confirms that membrane modification is chemically stable. Additionally, the reduction in the intensity of specific peaks associated with organic fouling (C–H and –OH groups) after SDS cleaning demonstrates the effectiveness of the cleaning process in removing adsorbed latex contaminants. Referring to Figure 1a, the pristine membrane exhibits distinctive peaks of PVDF at 1402, 1275, 1179, and 1070 cm^−1^ [21,22]. The observed peaks at 1400 cm^−1^ correspond to the deformation vibration of –CH_2_, while the symmetrical and asymmetrical stretching of –CF_2_ are associated with 1274 cm^−1^ and 1179 cm^−1^, respectively. One of the characteristic peaks of PVDF is detected at a wavenumber of 877 cm^−1^, while the stretching vibration of the –CH group is observed at 840 cm^−1^.

The bands seen within the spectral range of 800 to 1400 cm^−1^ were ascribed to the lattice vibrations of TiO_2_ in the composite material [23]. The peak at around 1065–1070 cm^−1^ indicates the existence of the –OH stretching vibration [24]. The membranes displayed a stretching vibrational peak of carbon monoxide (CO) at a wavenumber of 1650 cm^−1^, indicating the existence of residues [2,25]. Polyvinylpyrrolidone (PVP) with a molecular weight exceeding 10,000 g/mol has a higher tendency to be incorporated into the membrane [26,27]. The PVP spectra exhibited three clearly distinguishable peaks at 1290 cm^−1^, 1660 cm^−1^, and 1463 cm^−1^, which corresponded to the stretching vibrations of C-N, C=O, and CH_2_ bonds, respectively. The presence of PVDF, TiO_2_, and PVP in the membrane is shown by the peaks, as published by [10].

Figure 1a,b present the FTIR spectra of the PVDF–PVP–TiO_2_ membrane after filtration and after SDS cleaning. After 2 h of filtering the skim latex, the membrane was soaked in a 10% solution of sodium dodecyl sulfate (SDS) for one hour to eliminate the pollutants that were adhered to the surface of the membrane; then the membrane was reused for skim latex filtration after rinsing the membrane with deionized water. This membrane showed similar peaks to those of the PVDF-TiO_2_-PVP membrane before SDS cleaning.

The authors of [3] showed that the FTIR spectra of raw skim latex and concentrated skim latex films include functional groups in the range of 4000–2900 cm^−1^ to 1800–700 cm^−1^. The distinct absorption peaks of cis-1,4-polyisoprene at 835 cm^−1^, 1664 cm^−1^, and 2962 cm^−1^ correspond to the vibrations of C-H olefin wagging, C=C stretching, and C-H_2_ stretching, respectively [28]. Different from both fresh natural rubber and HANR films, the FTIR spectra of concentrated latex and raw skim latex were clearly visible [29]. The absorption peaks of the skim latex films exhibited broad and overlapping characteristics, especially in the areas of 1050–1100 cm^−1^, 1740–1600 cm^−1^, and 3100–3700 cm^−1^. The wide peaks were linked to the substantial presence of non-rubber components, specifically proteins and phospholipids. The presence of N-H and O-H bonds was attributed to the broad absorption peak that occurred between 3200 and 3600 cm^−1^. Skim latex films likely have a higher percentage of non-rubber components than rubber [30].

#### 3.1.2. FESEM-EDX of Membranes Post-Filtration

FESEM images provide critical morphological insight into the surface characteristics of PVDF-TiO_2_-PVP membranes post-skim latex filtration and after sodium dodecyl sulfate (SDS) cleaning. These observations are supported by prior compositional analyses and consistent with the reported literature on membrane fouling behavior during latex processing.

Based on a previous related study, the membrane containing 16 wt.% PVDF exhibited a surface accumulation of agglomerated skim latex particles post-filtration, indicating particle adhesion. An analysis of particle size, morphology, and distribution provides insights into filtration performance. Filtration using a PVDF-PVP-TiO_2_ membrane showed a tendency for latex particles to become entrapped on the surface [5].

**Figure 2:**
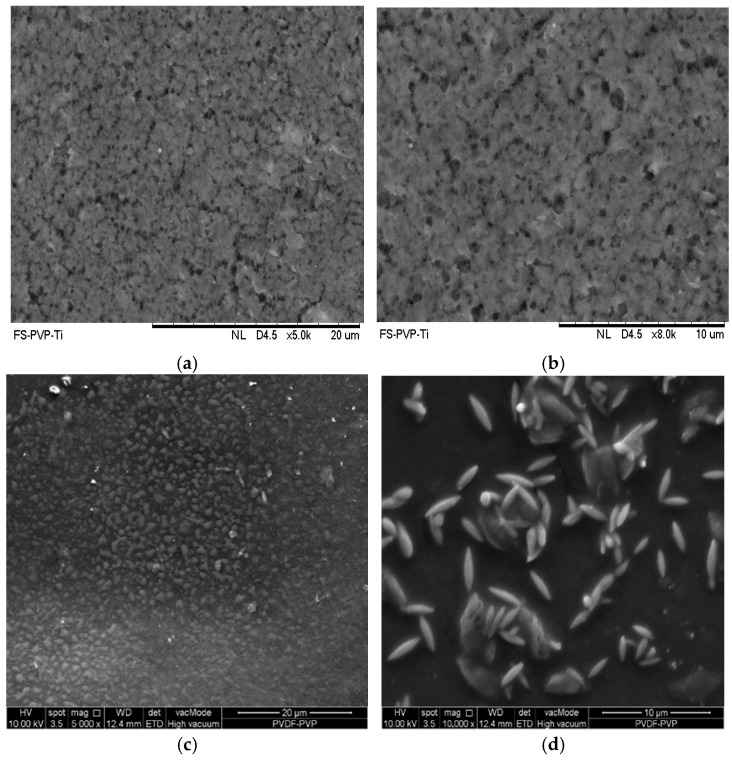
FESEM of (**a**) pristine membrane before filtration at 5000× magnification; (**b**) pristine membrane before filtration at 8000× magnification; (**c**) membrane post-skim latex filtration at 5000× magnification; (**d**) membrane post-skim latex filtration at 10,000× magnification.

Figure 2 shows the FESEM results of membranes with different magnifications and reveals an extensive accumulation of skim latex particulates on the membrane surface. As shown in Figure 2a,b, pristine membranes exhibit a smooth and homogeneous surface, confirming that no particle deposition or agglomeration is present before the filtration process. Figure 2c,d show that the particles exhibit irregular shapes and sizes, with a tendency to form both dispersed and localized agglomerates. Elliptical and rod-like structures, characteristic of natural latex microdomains, are strongly adhered to the membrane surface.

The presence of these agglomerates confirms substantial fouling, likely due to the physicochemical interactions between the latex proteins and the hydrophilic/hydrophobic domains of the membrane. As previously reported, membranes with 16 wt.% PVDF exhibit higher tendencies for surface fouling due to the entrapment of colloidal particles. Additionally, the combination of PVDF and PVP within the matrix provides a dual-phase environment conducive to selective adsorption and particle retention.

These observations align with the EDX results, which showed high carbon and sulfur contents, indicative of proteinaceous and rubber-derived components. The accumulation of latex particles impacts membrane permeability and highlights the importance of surface morphology in governing separation efficiency.

**Figure 3:**
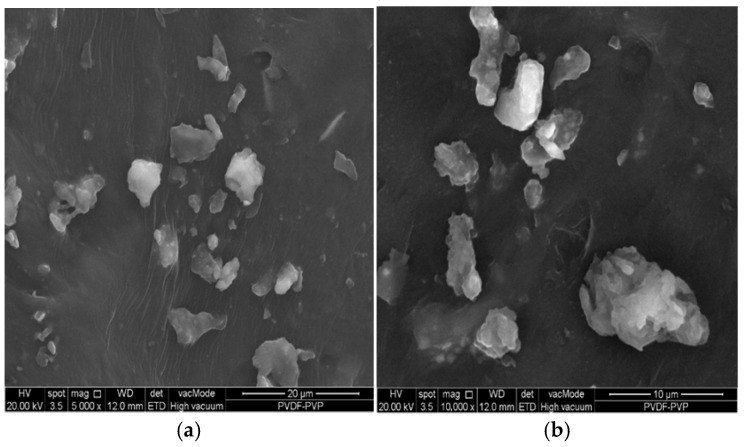
FESEM of membrane post-skim latex filtration after cleaning with SDS: (**a**) 5000× magnification; (**b**) 10,000× magnification.

Figure 3a,b show the membrane surface following SDS cleaning treatment. A noticeable reduction in surface fouling is observed, with fewer large agglomerates and a more exposed membrane texture. The remaining particles are scattered and significantly smaller in size, indicating the partial removal of the fouling layer.

SDS, an anionic surfactant, disrupts hydrophobic interactions between latex proteins and the membrane matrix, thereby promoting the desorption of foulants. This cleaning efficacy is also supported by the EDX analysis, which showed a marked decrease in carbon content (from 84.74 wt.% to 63.85 wt.%) and increased oxygen levels, suggesting oxidative surface modification or residual SDS presence. Notably, TiO_2_ particles embedded within the membrane are more apparent post-cleaning, as seen based on smoother, clearer surface zones, which were previously masked by fouling. The presence of trace elements (Si, Al, Fe) may be attributed to environmental contamination or remnant latex stabilizers.

Comparing pre- and post-cleaning images, it is evident that the SDS treatment effectively reduces fouling, restoring membrane surface properties critical for repeated use potential performance. The persistence of some particulate matter, however, suggests that a single SDS treatment may not fully regenerate the membrane, and multistep or enzymatic cleaning approaches may be required for complete foulant removal. The observations reinforce the need for membrane surface modifications to enhance antifouling properties potentially through surface functionalization or the incorporation of higher TiO_2_ content for photocatalytic self-cleaning. The UV-induced photocatalytic activation of TiO_2_ has been widely reported to generate reactive oxygen species (•OH radicals) capable of degrading organic foulants on membrane surfaces, thereby enhancing self-cleaning performance and fouling resistance [26].

**Figure 4:**
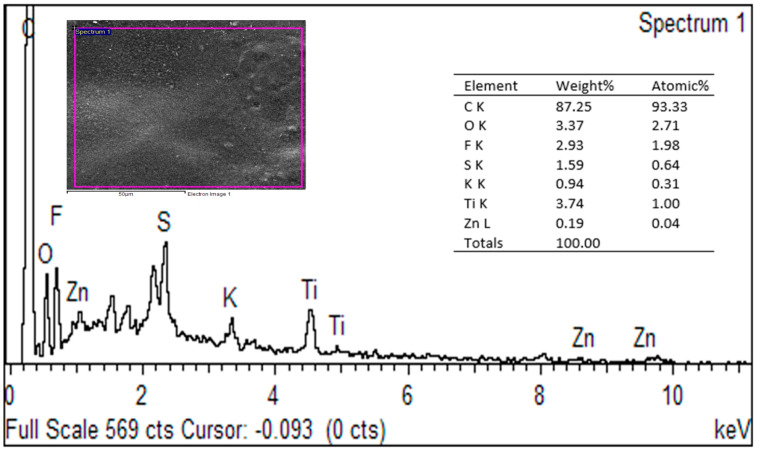
EDX of membrane post-skim latex filtration.

The energy-dispersive X-ray spectroscopy (EDX) spectrum presented provides insight into the elemental composition of the PVDF-TiO_2_-PVP mixed matrix membrane surface following skim latex filtration. The elemental analysis of the pristine membrane reveals the presence of C, O, F, and Ti, verifying the successful incorporation of TiO_2_ within the PVDF–PVP matrix. As presented in Figure 4, the dominant elements detected were carbon (C) and oxygen (O), accounting for 84.25 wt.% and 3.37 wt.%, respectively, corresponding to 93.33 at.% and 2.71 at.%. The high carbon content is consistent with the polymeric backbone of PVDF and PVP, primarily composed of carbon-based chains such as polyvinylidene fluoride and polyvinylpyrrolidone.

Skim latex, derived from natural rubber, primarily comprises hydrogen, carbon, and oxygen, with elemental composition varying by source and additives [5]. Elemental analysis revealed carbon as the dominant element, accounting for 94.46 wt.% and 99.16 at.%, consistent with its cis-1,4-polyisoprene polymer structure. EDX analysis also detected zinc, originating from additives such as ZnO, ZDEC, and ZMBT used in the rubber film manufacturing process [31]. Raw natural rubber consists predominantly of pure rubber (90–95%), with minor constituents including fatty acids (1–2%), proteins (2–3%), sugars (0.2–0.5%), and inorganic salts (0.5%), containing elements such as sodium, potassium, magnesium, phosphorus, calcium, copper, manganese, and iron [26].

The presence of titanium (Ti) at 3.74 wt.% (1 at.%) confirms the successful incorporation of TiO_2_ nanoparticles within the membrane matrix. These nanoparticles are known to enhance hydrophilicity and antifouling properties, playing a critical role in latex filtration performance. Minor elements such as fluorine (F, 2.93 wt.%, 1.98 at.%) are characteristic of the PVDF polymer, which contains fluorinated monomer units. Sulfur (S, 1.59 wt.%, 0.54 at.%) and potassium (K, 0.94 wt.%, 0.31 at.%) likely originate from the residual latex or chemical additives present during latex processing or membrane fabrication. Zinc (Zn, 0.19 wt.%, 0.04 at.%) may indicate remnants from common vulcanization activators in skim latex, such as zinc oxide or zinc-based accelerators.

The detected elemental profile supports the conclusion that latex components and membrane additives are retained on the membrane surface post-filtration. The entrapment of these substances suggests surface fouling due to latex particle adhesion, as also indicated by the SEM micrograph. The presence of inorganic residues, such as Ti and Zn, further supports the hypothesis of particle–membrane interaction facilitated by hydrophilic and catalytic components within the membrane.

**Figure 5:**
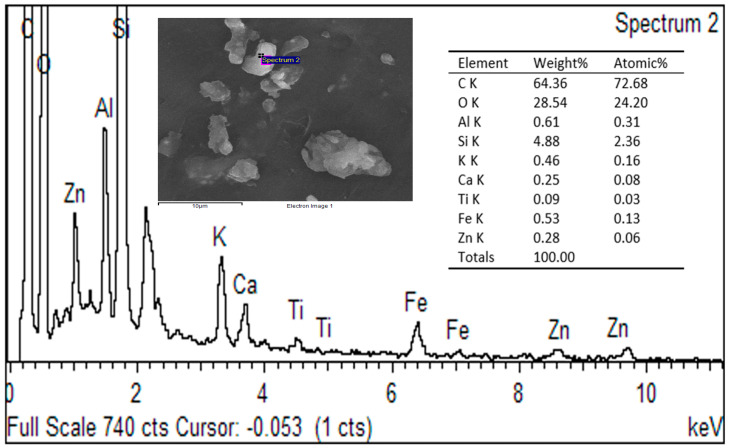
EDX of membrane post-skim latex filtration after cleaning with SDS.

Figure 5 illustrates the EDX of the membrane post-skim latex filtration after cleaning with SDS. The EDX spectrum enables an elemental analysis of the PVDF-TiO_2_-PVP membrane surface following skim latex filtration and subsequent cleaning with sodium dodecyl sulfate (SDS). The primary elements detected include carbon (C, 64.36 wt.%) and oxygen (O, 28.54 wt.%), which are indicative of the organic matrix of the membrane and residual components from the latex and cleaning agent.

The elevated oxygen content, significantly higher than in the unwashed membrane, suggests the effectiveness of SDS in removing organic contaminants while possibly leaving behind oxygen-containing surface groups or residues. SDS, an anionic surfactant, facilitates the desorption of organic fouling [27], contributing to the reduction in carbon content compared to the unwashed membrane (from 87.25 wt.% to 64.36 wt.%).

Silica (Si, 4.88 wt.%) and aluminum (Al, 0.61 wt.%) were also detected. These may originate from external contamination, such as environmental dust, or from equipment surfaces during cleaning or analysis. Potassium (K, 0.46 wt.%) and calcium (Ca, 0.25 wt.%) may reflect residual elements from latex stabilizers or additives, although their relatively low concentrations suggest effective partial removal during SDS treatment.

Traces of titanium (Ti, 0.09 wt.%) are consistent with the embedded TiO_2_ nanoparticles in the membrane matrix. The reduced signal relative to the unwashed membrane (3.74 wt.%) implies a potential surface masking effect due to the accumulation of residual substances pre-cleaning or Ti particles being less exposed post-cleaning. Iron (Fe, 0.53 wt.%) and zinc (Zn, 0.28 wt.%) were detected in low amounts and may derive from residual skim latex components or process equipment corrosion.

### 3.2. Membrane Performance and Membrane Cleaning Analysis

Skim latex serum, a by-product generated from the treatment of latex effluent in rubber processing industries, comprises a complex mixture of organic constituents, sulfates, ammonia, and various inorganic materials [32,33]. In the present work, skim latex was subjected to membrane-based ultrafiltration, resulting in the separation of two distinct fractions: a permeate stream (skim serum) and a retained phase (skim latex concentrate).

**Figure 6:**
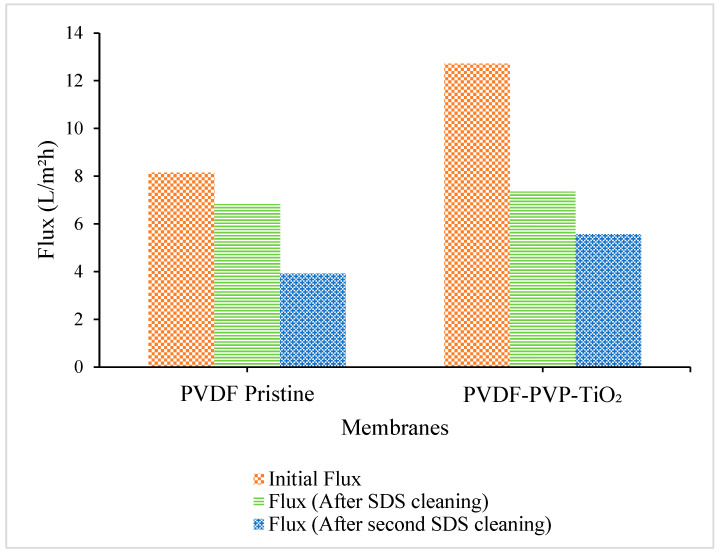
Skim serum flux (initial flux and flux after membrane cleaning with SDS agent).

Figure 6 presents the permeate flux performance of two membrane types (PVDF and PVDF/PVP/TiO_2_) measured at three different stages: the initial filtration cycle, after the first sodium dodecyl sulfate (SDS) cleaning, and following a second SDS cleaning cycle. The results provide insight into both the membranes’ initial productivity and their reusability and cleaning efficiency.

As shown in Figure 6, the PVDF-PVP-TiO_2_ membrane achieved the highest initial permeate flux of 12.72 L/m^2^h, significantly outperforming the pristine PVDF membrane, which exhibited an initial flux of 8.14 L/m^2^h. This improvement can be attributed to the enhanced hydrophilicity and antifouling properties imparted by the addition of PVP and TiO_2_ nanoparticles. PVP acts as a pore-forming agent and increases membrane porosity, while TiO_2_ contributes to surface hydrophilicity and may reduce foulant adhesion through its photocatalytic and surface-modifying effects [34,35].

Figure 6 illustrates that the membranes can be reused after cleaning with sodium dodecyl sulfate (SDS) and still offer good filtration performance for skim latex depending on several factors. The membranes’ reusability was further assessed through sequential cleaning cycles using sodium dodecyl sulfate (SDS), a common anionic surfactant effective at removing organic and colloidal fouling. After the first SDS cleaning, both membranes maintained good filtration performance. The PVDF pristine membrane recovered to 6.82 L/m^2^h, while the PVDF-PVP-TiO_2_ membrane recovered to a slightly higher flux of 7.35 L/m^2^h. This indicates that SDS was effective in removing reversible foulants and restoring operational flux. The higher flux recovery in the MMM is likely due to the increased surface hydrophilicity, which reduces fouling strength and enhances foulant detachment during cleaning.

Nevertheless, after the second SDS cleaning, a decline in membrane performance was observed in both cases. After the second SDS cleaning cycle, the membrane could still be used, but the flux dropped to 3.91 L/m^2^h for the PVDF pristine membrane and declined to 5.57 L/m^2^h for the PVDF-PVP-TiO_2_ membrane. This reduction suggests the presence of irreversible fouling or the structural degradation of membrane pores, particularly in the pristine PVDF membrane, which is more susceptible to long-term fouling due to its hydrophobic nature. In contrast, the PVDF–PVP–TiO_2_ membrane demonstrated greater resistance to flux decline, indicating improved long-term stability and reusability potential.

These results confirm that membrane modification with PVP and TiO_2_ enhances both initial performance and long-term operability, making the MMM a promising candidate for repeated use ultrafiltration applications in natural rubber processing. Furthermore, while SDS cleaning is effective, its efficiency diminishes over successive cycles, highlighting the need for optimizing cleaning protocols or exploring advanced cleaning strategies for sustainable membrane reuse.

The concentration of materials near the membrane surface restricted some pore blocking events, and the tiniest chemicals that entered the pores and were present in the permeate had no meaningful interactions with the membrane. Such dominant exterior fouling appears to be a big favorable factor because of its reversibility to boost latex suspension concentration via filtration on a porous membrane [1].

Flux can also be influenced by the membrane material used and its properties. Pristine PVDF membranes may be less resistant to fouling and particle deposition than modified membranes, such as PVDF-TiO_2_ with PVP. The poor flow observed in skim latex filtering using membranes can be attributed to a variety of factors, including skim latex particle deposition and fouling [7,8]. Fouling is a significant barrier to the implementation of membrane separation methods. Ensuring the cleanliness of membranes decreases fouling and enhances membrane function. Efficient cleaning procedures have become essential for membrane separation operations in water treatment [8]. The process of particles, macromolecules, proteins, salts, colloids, and other contaminants accumulating on a membrane’s surface or within its pores is known as fouling. Current research mostly focuses on the study of organic matter fouling in oil, including algae, proteins, colloidal particles, and other organic substances [27,31]. It is essential to characterize the foulants to determine whether they accumulate on the membrane’s surface or inside the pores. Several factors influence fouling, including temperature, crossflow velocity, transmembrane pressure, roughness, pore size, feed pH, shape, the concentration of foulants, the size of foulants, and the hydrophilicity or hydrophobicity of the membrane [3,36,37]. Fouling is the accumulation of fluid particles or chemicals on the surface or inside the pores of a membrane, leading to a reduction in its permeability. The accumulation of proteins, lipids, and other constituents of skim latex on the membrane surface might result in fouling during the filtration process. Reduced flux is the result of the fouling layer blocking fluid flow [38].

Madaeni et al. [24] found that PVDF microfiltration membranes can be chemically cleaned to remove milk proteins. Cleaning time and cleaner concentration were among the cleaning characteristics that were examined. According to their research, the efficiency of the cleaner was affected by its concentration. Another study found that a higher concentration of cleaner improves cleaning efficiency for both acidic and alkaline solutions. They asserted that this also pertains to surfactants. This study used sodium dodecyl sulfate (SDS) anionic surfactant as the cleaning agent [8]. The backwash flow helped to transport the foulant away from the membrane surface, facilitating its removal [24].

Organic foulants such as proteins and lipids are known to interact strongly with hydrophobic polymeric surfaces, including fluorinated polymers like PVDF, primarily through hydrophobic interactions and adsorption mechanisms, which significantly contribute to membrane fouling behavior.

Recent studies have demonstrated that TiO_2_-integrated PVDF membranes can effectively mitigate fouling through the photocatalytic degradation of organic contaminants under UV irradiation, enabling significant flux recovery and enhanced antifouling performance [39,40]. Furthermore, advanced photocatalytic membrane systems have shown that the in situ degradation of adsorbed organic foulants can restore permeability and improve long-term operational stability, highlighting the potential of TiO_2_-based catalytic cleaning strategies [41].

The features of a membrane such as thickness, porosity, and the composition of the membrane determine its susceptibility to cleaning agents. Skim latex properties such as concentration, particle size, and the composition of skim latex can affect adsorption onto the membrane and ease of cleaning. Some potential benefits of reusing a membrane are cost-effectiveness and environmental sustainability. Reusing a membrane reduces material costs compared to constant replacements and results in less waste generation compared to disposable membranes. Reusing a PVDF-TiO_2_-PVP membrane after cleaning with SDS as the anionic surfactant is possible but requires a careful consideration of cleaning methods, membrane characteristics, and desired filtration performance. Evaluating membrane performance after cleaning and optimizing cleaning protocols are crucial for successful reuse.

## 4. Conclusions

The comprehensive characterization of the PVDF-PVP-TiO_2_ mixed matrix membrane (MMM) through FTIR, EDX, and FESEM analyses provides compelling evidence of its chemical stability, antifouling capability, and potential for repeated use in skim latex ultrafiltration. The FTIR results affirm the structural integrity of the membrane throughout the filtration and cleaning processes, with no significant alterations to the functional groups of PVDF and PVP, indicating robust chemical durability. EDX analysis further reveals a notable reduction in surface carbon content and an increase in oxygen levels after SDS cleaning, suggesting the effective removal of organic foulants. However, the presence of trace elements such as Si, Al, K, and Zn may point to either residual impurities or external contamination, warranting further investigation. The observed decrease in surface titanium intensity post-cleaning could be attributed to either surface coverage by residual compounds or the redistribution of TiO_2_ particles. FESEM imaging highlights substantial membrane fouling characterized by latex particle agglomeration, which is partially alleviated following SDS treatment. While SDS cleaning demonstrably improves membrane surface conditions, its effectiveness diminishes over multiple cycles, suggesting limitations in its long-term cleaning efficacy. Collectively, these findings emphasize the significance of membrane surface engineering in controlling fouling behavior and enhancing operational performance. The incorporation of PVP and TiO_2_ into the PVDF matrix not only improves initial flux and antifouling properties but also supports structural resilience under repeated cleaning. Therefore, the developed MMM exhibits strong potential for sustainable ultrafiltration applications in the natural rubber industry. Nonetheless, optimizing cleaning protocols or integrating alternative cleaning strategies remains essential to ensure prolonged membrane lifespan and cost-effective operation in industrial settings.

## Data Availability

The original contributions presented in this study are included in the article. Further inquiries can be directed to the corresponding authors.

## Abbreviations

The following abbreviations are used in this manuscript:

PVDF	Polyvinylidene fluoride
PVP	Polyvinylpyrrolidone
TiO2	Titanium dioxide
SDS	Sodium dodecyl sulfate
FESEM	Field emission scanning electron microscope
EDX	Energy-dispersive X-ray spectroscopy
FTIR	Fourier-transform infrared spectroscopy
MMMs	Mixed matrix membranes
